# Effect of Industrial By-Products on Unconfined Compressive Strength of Solidified Organic Marine Clayey Soils

**DOI:** 10.3390/ma8085098

**Published:** 2015-08-07

**Authors:** Chan-Gi Park, Sung-Wook Yun, Phillippe C. Baveye, Chan Yu

**Affiliations:** 1 Department of Rural Construction Engineering, Kongju National University, Yesan 143-701, Korea; E-Mail: cgpark@kongju.ac.kr; 2 Institute of Agriculture & Life Science, Gyeongsang National University, Jinju 660-701, Korea; E-Mail: wook@gnu.ac.kr; 3 Laboratory of Soil and Water Engineering, Department of Civil and Environmental Engineering, Rensselaer Polytechnic Institute, 110 8th street, Troy, NY 12180, USA; E-Mail: baveyp@rpi.edu; 4 Department of Agricultural Engineering, Gyeongsang National University, Jinju 660-701, Korea

**Keywords:** organic marine clayey soils, solidification agents, admixture, steelmaking slag, unconfined compressive strength

## Abstract

The use of industrial by-products as admixture to ASTM Type I cement (ordinary Portland cement (OPC)) was investigated with the objective of improving the solidification of organic marine clayey soils. The industrial by-products considered in this paper were oyster-shell powder (OSP), steelmaking slag dust (SMS) and fuel-gas-desulfurized (FGD) gypsum. The industrial by-products were added to OPC at a ratio of 5% based on dry weight to produce a mixture used to solidify organic marine clayey soils. The dosage ratios of mixtures to organic marine clayey soils were 5, 10 and 15% on a dry weight basis. Unconfined compressive strength (UCS) test after 28 days revealed that the highest strength was obtained with the OPC + SMS 15% mixing ratio. The UCS of specimens treated with this mixture was >500 kPa, compared with 300 kPa for specimens treated with a 15% OPC + OSP mixture and 200 kPa when 15% of OPC was used alone. These results were attributed to the more active hydration and pozzolanic reaction of the OPC + SMS mixture. This hypothesis was verified through X-ray diffraction (XRD) and scanning electron microscopy (SEM) analyses, and was confirmed by variations in the calcium carbonate (CaCO_3_) content of the materials during curing.

## 1. Introduction

Coastal mudflats (or estuarine sediments) are deposited in estuarine or river-mouth areas and are generally composed of clay, silt, calcareous shell debris, and organic matter. They have been recognized as weak grounds and are therefore not appropriate for building structures [1]. However, there is currently a need to develop harbors and other civil works, such as roads or railroads, upon such weak organic marine clayey materials. 

Traditional treatment strategies for these areas tended to be overly time-consuming and expensive, and inevitably disturbed the ecology of the tidal lands. Solidification methods have recently become more popular because they improve the mechanical properties of the weak ground in a short time period, while causing minimal damage to the ecological systems at the sites [2,3,4,5]. 

The solidification method was developed in the late 1960s in Sweden and Japan, and involves mixing solidification agents with weak marine sediments (mainly organic clayey soils) [6]. ASTM Type I cement (ordinary Portland cement (OPC)) is now commonly used as a solidification agent in a slurry form (wet method) [6,7]. The higher the solidification agent content, the greater the amounts of calcium silicate hydrates (C–S–H) and calcium hydroxide (Ca(OH)_2_) that are available and, hence, the greater the amount of calcium carbonate (CaCO_3_) that eventually results from carbonation. CaCO_3_ forms from the reaction of carbon dioxide (CO_2_) with Ca(OH)_2_ during the pozzolanic reaction and hardening process.

Organic marine clayey soils contain very fine particles, have considerable impurities and have high moisture contents. It is accepted that a high OPC addition ratio (over 20% based on the sediment dry weight) is required to achieve the treatment goal. Admixtures, such as silica or lime, can be used to reduce the addition ratio of OPC and enhance the treatment efficiency [8,9,10]. Other industrial by-products with high calcium contents and low cost could also be attractive alternative admixtures [11,12,13,14]. Additionally, significant economic benefits could be gained by replacing a substantial part of the OPC with inexpensive industrial by-products; greenhouse gas emissions associated with OPC production could be alleviated, and large amounts of industrial or societal waste could be recycled into durable underground construction materials [15]. 

In this general context, the primary objective of this study was to examine the potential of industrial by-products as alternative solidification agents for organic marine clayey soils. Unconfined compressive strength (UCS) tests were used to evaluate solidification effects with industrial by-product use. Changes in chemical properties and microstructural development during curing were also investigated.

## 2. Materials and Methods

### 2.1. Materials

A representative sample was selected at Bosung County, Chunnam Province, approximately in the middle between the western and eastern coasts of Korea. Because estuary sediments in Korea have similar physical and chemical characteristics due to having the same geological depositional units, the succession as a typical Holocene to pre-Holocene tidal-flat sedimentary up to 20 m depth [15,16]. The organic matter content of the sample was 5.87% (Table 1). The natural water content of the sample was not much lower than its liquid limit (LL) water content. The ratio of the LLs between the as-sampled and the oven-dried sample was <0.75 and, hence, it was classified as organic high plasticity soil by Unified Soil Classification System. The pH and electrical conductivity (EC) were determined in 1:5 (w/v) soil water suspensions using a Orion 550A pH meter (Thermo Fisher Scientific, Inc., Waltham, MA, USA). The pH value of hydration reaction of OPC concrete mass is above 12 even though it must be lower than that for soils.

**Table 1:** Physical and chemical properties of organic marine clayey soil.

Item	Value	Item	Value
Gs ^1^	2.44	Plasticity Index ^2^ (%)	16.67
Natural moisture content (% by mass)	60.2	Organic Content (%)	5.87
Liquid Limit (%)	68.89	pH	8.3
Plasticity Index (%)	21.1	Electric Conductivity (dS/m)	2.46
Liquid Limit ^2^ (%)	51.29	-	-

^1^ specific gravity of soil sample; ^2^ result after oven drying.

In this study, the industrial by-product materials of oyster-shell powder (OSP), steelmaking slag dust (SMS) and flue-gas-desulfurized gypsum powder (FGD) were explored as alternative admixtures to OPC. The OSP was produced through processes of “calcining (at 900 °C)” and “grinding.” The former consists of burning out impurities remaining on the surface of oyster-shell. SMS was produced during the separation of molten steel from impurities in steel-making electrical arc furnace (EAF). New SMS was stored and “cured” for about six months, and then was crushed, sized, and screened for sale for many different uses, *i.e.*, subbase of road, fill material, surcharge load in earth work, *etc.* During these procedures, the powder (or dust) was collected. FGD is a unique synthetic product derived from flue gas desulfurization systems at coal-fired furnace system.

**Figure 1:**
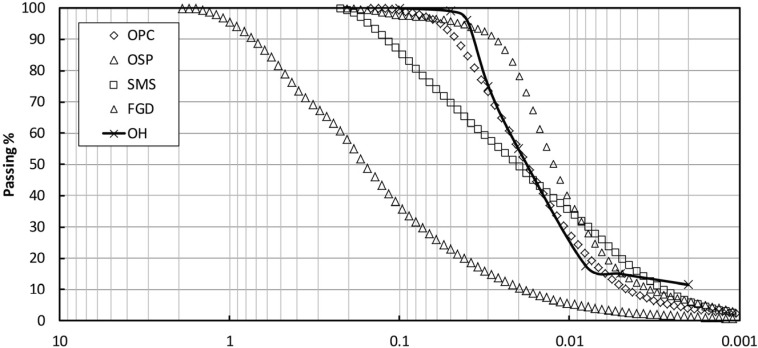
Particle size distribution of the different materials.

Particle size distributions of the various materials were determined with a CILAS (Orleans, France) laser diffraction particle size analyzer (Figure 1). FGD was finer than the sediment and OSP was coarser than the sediment; however, the particle size distributions of OPC and the sediment were similar.

X-ray fluorescence (XRF; Model XRF-1800/WD, Shimadzu Co., Kyoto, Japan) analyses of the OPC and admixtures (Table 2) suggests that FGD had a high Ca content (> 64.9%) while that of OSP was lower (46.4%). Processed OSP is generally a component of CaO, with about 97% total CaO composition, and often applied as an additive of OPC [17]. SMS had a much lower Ca content (24.7%) but had high Si and Fe and relatively high Al and Mn contents. The mineralogical contents of the soil and admixtures were determined by XRD (Model D8 Advance, Bruker AXS, Karlsruhe, Germany). The analyzed material was ground to a powder so that it completely passed through a No. 200 (75 μm) sieve. 

**Table 2:** Chemical properties and X-ray fluorescence (XRF) analysis results of admixtures. OPC: ordinary portland cement; OSP: oyster-shell powder; SMS: steelmaking slag dust; FGD: fuel-gas-desulfurized.

Materials	pH	EC (dS·m^−1^)	Blaine Finess (cm^2^/g)	Main Elements Contents (%) by XRF								
				Ca	O	Si	Fe	Mg	Al	S	Cl	LOI ^♯^
OPC	12.1	7.50	2800	44.99	35.22	10.37	2.03	2.01	1.86	1.51	-	1.96
OSP	8.7	0.61	3843	46.43	48.08	1.77	0.31	-	0.56	0.30	0.83	2.12
SMS	12.0	4.95	4500	24.74	33.92	8.63	21.02	2.81	3.06	0.44	0.15	1.88
FGD	12.7	7.98	3800	64.84	-	1.45	1.65	1.37	0.38	16.17	11.53	2.49

**^♯^** Loss on ignition.

**Figure 2:**
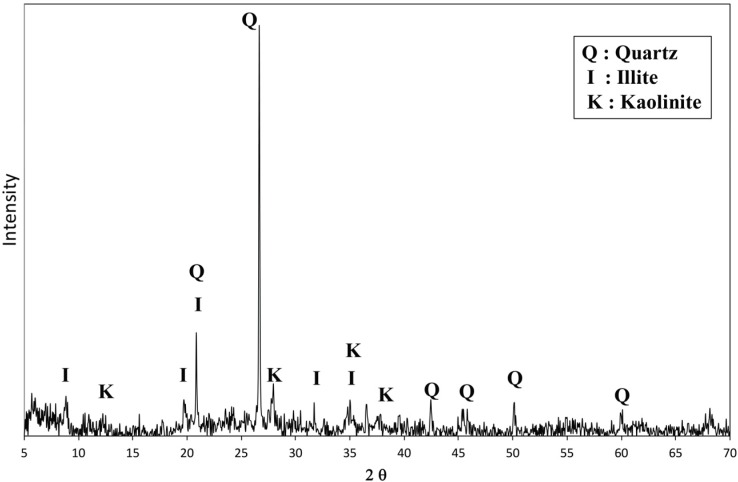
X-ray diffraction (XRD) diffractograms of organic marine clayey soil.

**Figure 3:**
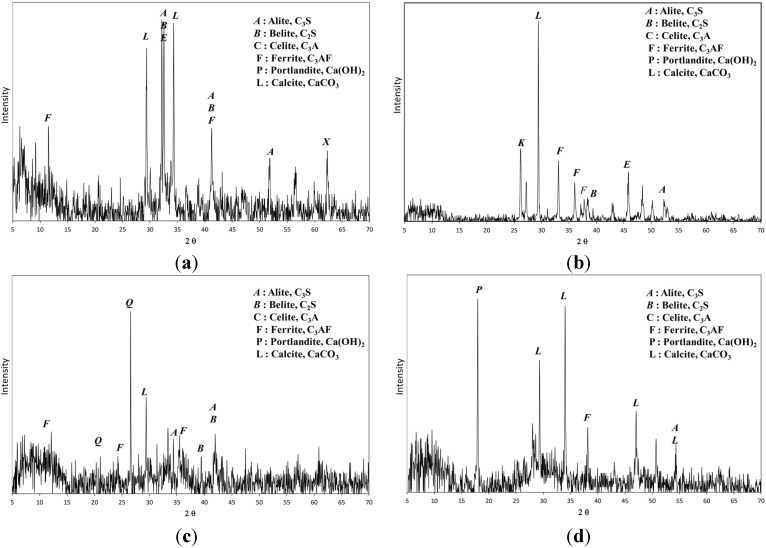
XRD diffractograms of ordinary Portland cement (OPC) and admixtures (**a**) OPC; (**b**) Oyster-shell powder (OSP); (**c**) Steelmaking slag dust (SMS); (**d**) Fuel-gas-desulfurized (FGD).

In the XRD diffractograms for the sediment sample (Figure 2), illite and kaolinite appeared to be the dominant clay minerals; quartz (SiO_2_) was also identified. OPC consisted primarily of four minerals, *i.e.*, alite (C_3_S), belite (C_2_S), celite (C_3_A) and ferrite (C_4_AF) [18,19]. The XRD diffractogram of OSP (Figure 3b) indicates that it contained ferrite, calcite with alite and belite. The SMS contained alite, belite, celite and other minerals; the latter were similar to those found in OPC (Figure 3c). The FGD contained portlandite and calcite with alite, belite and celite. On the basis of the XRD data, it was expected that SMS would be a good solidification material because of its finer particle distribution and similar chemical constituents to OPC. 

Extraction tests were also carried out to verify the environmental safety of the admixture materials according to a Korean standard [20]. Extraction solution (diluted HCl solution by distilled water at pH = 5.8–6.3) was added to samples (<5 mm size) to collect soluble contaminants and was shaken for 6 h at 2000 rpm. The extracted solution was then filtered through a 0.45 μm filter and concentrations were determined using an inductively coupled plasma optical-emission spectrometer (ICP-OES, ELAN DRC II, Perkin Elmer, Waltham, MA, USA).

Table 3 shows the test results on admixtures as well as soil sample (OH). None of the materials presented concentrations that exceeded permitted levels.

**Table 3:** Results of heavy metal concentration test.

Material	Heavy Metal Concentration (mg/L)				
	Cu	Cd	Pb	Cr	As
Standard of Korea	1.0	0.3	3.0	1.5	1.5
OH	0.031	N.D.	0.003	0.004	N.D.
OPC	0.001	N.D.	N.D.	0.729	N.D.
OSP	0.085	N.D.	0.004	0.004	0.007
SMS	0.004	N.D.	N.D.	0.007	N.D.
FGD	N.D.	N.D.	N.D.	0.013	N.D.

N.D.: Non detected.

### 2.2. Mixing and Manufacturing of Test Specimens

Test specimens were prepared by mixing the organic marine clayey soil with OPC and the admixture materials. Admixture materials were first dried and then added to OPC based on the dry weight at a ratio of 5% to produce the solidification agents. Most patented solidification agents, which are OPC-based systems, have been used at the 5% mixing ratio [21]. This mixing ratio was also used in this study to facilitate comparisons with previous work. The solidification agent was mixed with organic marine clayey soils but generally not directly; *i.e.*, dry-type mixing. Rather, it was mixed with organic marine clayey soils as a slurry; *i.e.*, wet-type mixing. A slurry of the solidification materials was prepared by mixing it with fresh water at a desired ratio. 

The water-mixture ratio typically used is 0.8–1.2 for deep mixing, but is reduced for shallow mixing [21]. A water-mixture ratio of 1:1 was used in this study. The dosage ratio of the solidification materials to organic marine clayey soil was 5%, 10% or 15% on a dry weight basis. The mixtures (solidification agents + organic marine clayey soil + water) were mixed by hand for *ca*. 5 min. After mixing of the mixture, the duplicate specimens were molded for UCS test with curing day and mixing ratio using a cylinder mold (cylindrical PVC mold, 50 mm in diameter with an aspect ratio of 2.0). The mixture was cast into the mold and cured in a humidity chamber (23 ± 1 °C and relative humidity >96%) for 24 h and then extruded from the mold using a hydraulic jacking device; the samples, then, were sunk in the sea water tube for 7, 14 or 28 days (23 ± 1 °C and seawater) prior to strength testing. This curing condition was appropriate as they reflect conditions typically employed on local sites where treated soils are exposed under the seawater level.

### 2.3. Test Methods

#### 2.3.1. Unconfined Compressive Strength

UCS test was conducted at a strain rate of 1 mm/min according to the ASTM C 39–96 standard [22]. Each test was performed after 7, 14 and 28 days of curing.

#### 2.3.2. Optical Observations

Test involving pH, XRD and SEM (Model JEM–2010; JEOL Ltd., Tokyo, Japan) analyses, which were carried out to investigate the differences between the solidification materials with different types of admixture materials. 

#### 2.3.3. The Content of CaCO_3_

The calcium content of the mixtures was assumed to derive from the products associated with the hydration reaction. The EDTA titration method was used as follows [23]. Subsamples taken from the mixtures were dried at 105 °C for 24 h, cooled and weighed. The dry mixtures were then rinsed with a 0.1 N H_2_SO_4_ solution to allow the precipitates to dissolve into the liquid phase. The sample plus solution was mixed by hand for 10 min and filtered through 0.45 μm filter paper, after which the calcium content was determined by EDTA titration. 

## 3. Results and Discussion

### 3.1. Unconfined Compressive Strength 

**Figure 4:**
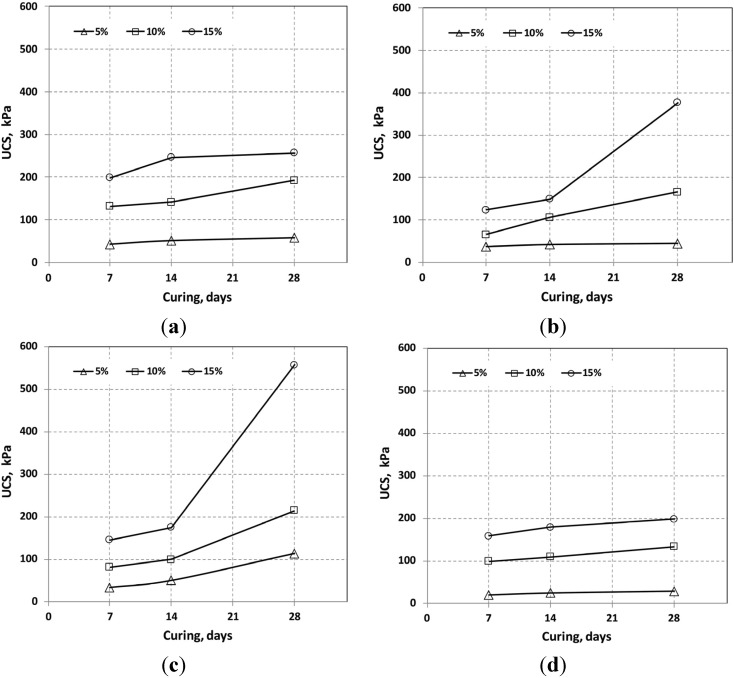
Relationship between curing days and unconfined compressive strength (UCS) of mixtures with admixtures. (**a**) OPC alone; (**b**) OPC + OSP; (**c**) OPC + SMS; (**d**) OPC + FGD.

**Figure 5:**
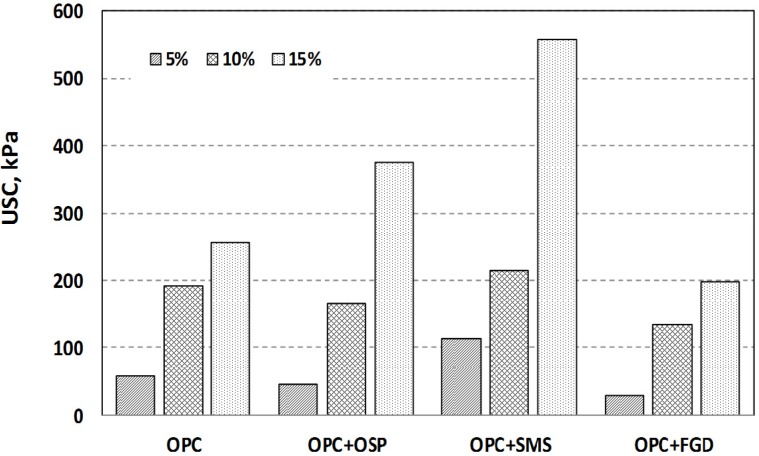
UCS with mixture with type of admixtures at 28 curing days.

**Figure 6:**
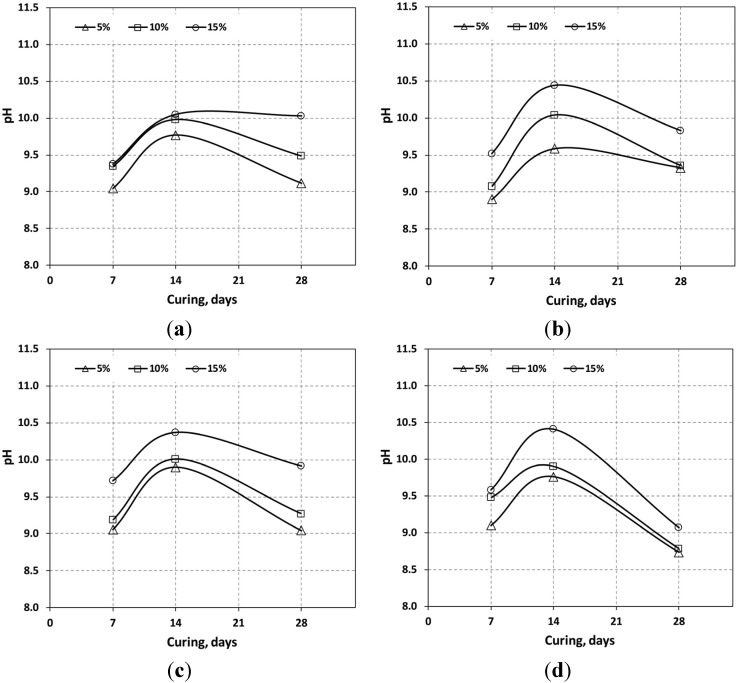
The pH values of mixtures with the curing time (**a**) OPC; (**b**) OPC + OSP; (**c**) OPC + SMS; (**d**) OPC + FGD.

The experimental results are summarized in Table 4 and shown graphically in Figure 4, Figure 5, Figure 6. The UCSes of OPC + SMS and OPC + OSP were higher than the UCS of OPC alone and OPC + FGD at 28 curing days. The UCSes of OPC alone were higher at initial curing stage than other cases, but at a mixing ratio of 15% content, the situation was reversed at 28 days of curing. It is effective in the strength development when added to an appropriate amount of pozzolanic material. Insufficient or excessive pozzolanic material would limit strength development [24,25]. The pozzolan reaction was activated by the increased OPC + OSP and OPC + SMS content at 15% mixing ratio at 28 days of curing. 

The performance with OPC + FGD was mediocre: the UCS at a mixing ratio of 15% was <200 kPa at 28 curing days. It is presumed that some interference prevented the FGD reaction from occurring with the OPC or sediment particles. On the other hand, FGD decreases the early hydration rate of cement-based materials and prolongs the setting time, thus requiring long curing times to achieve a high UCS [26,27].

The addition of quicklime to OPC is generally acknowledged to help counteract the high moisture content of sediments that affects the hardening process of OPC. Quicklime added to sediments modifies the fine particles, and OPC binds the modified peds of fine particles and thereby provides a degree of long-term strength gain because of enhanced pozzolanic reactions [26,27]. However, such phenomena were not clearly observed in this study, especially in the case of OPC + FGD. Furthermore, evidence in this respect was not strong at a low mixing ratio. A measureable improvement in UCS was observed under these conditions only with OPC + SMS at the 5% mixing ratio after 28 curing days.

**Table 4:** Results of experiment with the curing period of test specimens. UCS: unconfined compressive strength.

Curing (days)	Additive	Mixing Ratio	pH	Dry Density (g/cm^2^)	UCS (kPa)
7	OPC	5%	9.05	1.06	42.6
		10%	9.35	1.06	131.2
		15%	9.38	1.01	198.8
	OPC + OSP	5%	8.90	1.02	36.9
		10%	9.08	1.01	65.1
		15%	9.52	0.99	123.2
	OPC + SMS	5%	9.06	1.02	33.9
		10%	9.19	1.00	81.6
		15%	9.72	1.03	145.6
	OPC + FGD	5%	9.10	1.04	20.0
		10%	9.48	1.04	98.5
		15%	9.58	1.04	158.1
14	OPC	5%	9.77	1.04	51.0
		10%	9.98	1.05	140.9
		15%	10.05	1.03	245.9
	OPC + OSP	5%	9.59	1.02	41.8
		10%	10.04	1.02	106.2
		15%	10.44	1.01	148.8
	OPC + SMS	5%	9.90	1.02	50.6
		10%	10.01	1.03	99.3
		15%	10.37	1.05	174.4
	OPC + FGD	5%	9.76	1.03	24.6
		10%	9.90	1.02	109.2
		15%	10.41	1.05	179.0
28	OPC	5%	9.12	1.02	57.8
		10%	9.49	1.02	191.9
		15%	10.03	1.04	256.7
	OPC + OSP	5%	9.33	1.01	44.5
		10%	9.36	1.04	165.8
		15%	9.83	1.04	376.1
	OPC + SMS	5%	9.04	1.02	113.5
		10%	9.27	1.05	214.1
		15%	9.92	1.02	556.7
	OPC + FGD	5%	8.73	1.05	29.0
		10%	8.78	1.03	133.3
		15%	9.07	1.05	198.4

The UCS results for specimens mixed with OPC + OSP and OPC + SMS were expected, showing UCS increase rate values that were 50%–100% higher than the results demonstrated by OPC after 28 days of curing. This enhancement was attributed to the addition of the calcium component (mainly lime) in OSP and SMS, which led to a relatively slow strengthening of the mixtures because of the slow lime reaction kinetics. The higher UCS with OPC + SMS than with OPC + OSP was also attributed to the finer particle distribution of the former mix.

The UCS for the 15% mixing ratio of OPC + FGD was 198.4 kPa at 28 curing days. The Japanese Cement Association (1994) requires a surcharge of 150 kPa to improve soft ground. Our data indicate that the addition of 10% OPC, OPC + OSP or OPC + SMS is sufficient to meet the UCS requirement for the movement of heavy equipment in fields. Additionally, the higher UCS with OPC + SMS means that it could be used with the pavement subgrade method. 

### 3.2. pH Variation

The pH test results are shown in Figure 6. The pH increased up to 14 curing days and then decreased afterwards until the 28th curing day. This happened in most cases, with the exception of the sample involving 15% OPC; in this case, the pH did not change after 14 days. With OPC + OSP and OPC + SMS, the pH increased up to 14 curing days and then decreased until the 28th day. OSP and SMS mixtures had a similar range of pH values, *i.e.*, 9.3–9.8 and 9.0–9.9, respectively. The reduction in pH after 14 days of curing was more pronounced for the OPC + FGD mixture. When cementitious material is immersed in water, the alkaline component leaches out and the pH increases. However, the pH decreases with increasing age so that the decreased alkaline component leaches out and an increase in carbonate occurs [28].

### 3.3. Optical Observations

**Figure 7:**
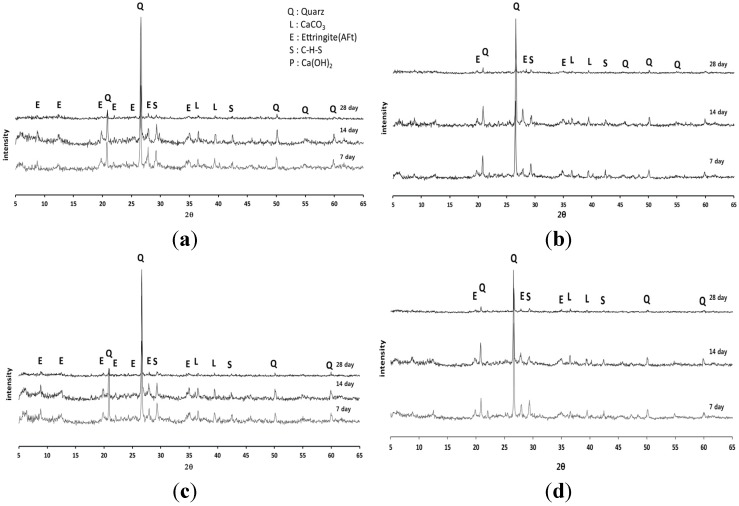
Comparison of XRD diffractogram of mixtures with the types of admixtures at 15% mixing ration of solidification agents. (**a**) OPC; (**b**) OPC + OSP; (**c**) OPC + SMS; (**d**) OPC + FGD.

Optical analysis was conducted on the specimens made with SMS that had the highest UCS. This technique is often applied to solidified samples to assess changes in the mixture components with solidification materials or as a function of curing time. XRD and SEM analyses were carried out to compare the changes in the components of the 15% mixing ratio mixtures, which were the most greatly affected by the admixture materials (Figure 7). 

The XRD diffractograms of all mixtures displayed prominent reflections for C–S–H, calcite and ettringite for the OPC + OSP (Figure 7b) and OPC + FGD (Figure 7d) mixtures. The diffractogram of the OPC + SMS mixture was similar to that of OPC and could be predicted from the XRD data of the individual admixture materials (see Table 2). Even at the lowest calcium oxide content among admixture materials, the observed reflections for the OPC + SMS system are evidence that hydration and the pozzolanic reaction were more active in the case of this combined system than in the other examples. 

**Figure 8:**
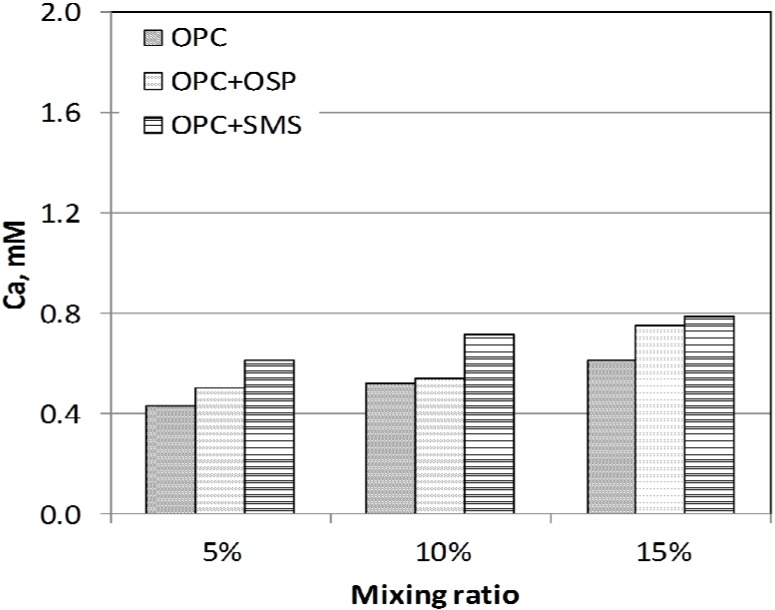
Ca content of mixtures with type of admixtures after 28 days.

Alkalis act as catalysts in the formation of calcium silicate hydrate from calcium oxide and silica. The calcium contents of the OPC alone, OPC + OSP and OPC + SMS mixtures after 28 days of curing were measured by EDTA titration (Figure 8). There was a good correlation between the UCS and the calcium content. C–S–H gel and portlandite amount to >75% of the hydrated OPC paste during the setting process and lead to either development of strength or its enhancement. 

**Figure 9:**
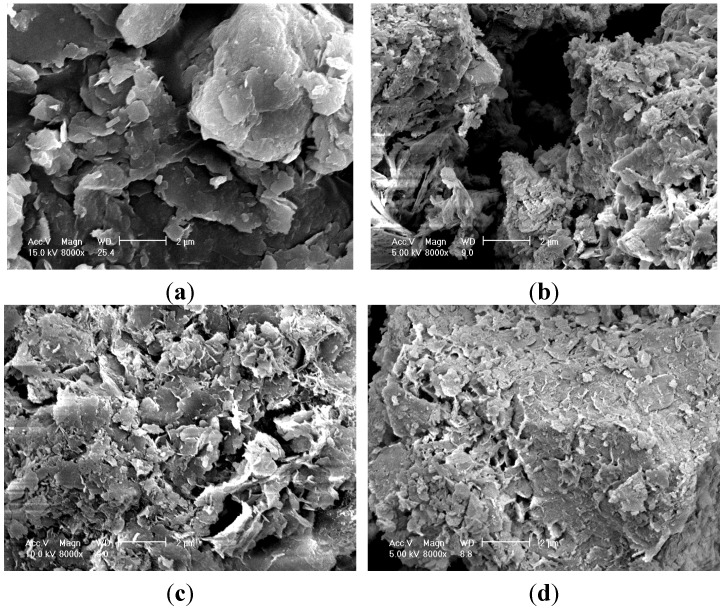
Scanning electron microscopy (SEM) photograph of organic clayey soil and mixtures with different types of admixtures. (**a**) Sediment; (**b**) OPC; (**c**) OPC + OSP; (**d**) OPC + SMS.

Ettringite also forms during the pozzolanic reaction and fills the pore spaces between particles. Therefore, the higher the calcium concentration, the higher the USC that can be expected for the mixture. However, the UCS for 5% OPC alone was larger than that for 5% OPC + OSP (Figure 5), but the calcium content for 5% OPC was smaller than that for 5% OPC + OSP (Figure 7). These results were attributed to insufficient mixing and possible compaction; however, this was a relatively small effect. SEM images (Figure 9) revealed fine platelets in the sediment (organic clay) that contained the Si and Al atoms corresponding to the kaolin detected by XRD analysis; the Si, Al and K corresponded to illite. There were no large or round minerals. There were some voids between the peds’ hydrated sediments for the OPC and OSP mixtures, but almost all of the voids were filled in the SMS case, giving the appearance of higher density.

## 4. Conclusions

The utility of industrial by-product materials as alternative solidification materials to OPC to improve the UCS of organic marine clayey sediments was explored. OSP, SMS and FGD were evaluated as alternative admixture materials. 

The highest strengths were achieved with a 15% mixing ratio of OPC + SMS; the UCS exceeded 500 kPa after 28 days of curing. This compared with *ca*. 200 kPa for OPC alone and 300 kPa with the OPC + OSP mixture. OSP and SMS could therefore be used as alternative admixture materials to OPC to improve the solidification efficacy of organic marine clays instead of the expensive conventional admixture materials. 
